# Expression of tissue factor pathway inhibitor-2 in gastric stromal tumor and its clinical significance

**DOI:** 10.3892/etm.2013.1448

**Published:** 2013-12-13

**Authors:** GUI-JUN WANG, YU-BIN WANG, DI-NUO LI, CHEN LI, BEI-BEI DENG

**Affiliations:** 1 Department of General Surgery, The First Affiliated Hospital, Liaoning Medical University, Jinzhou, Liaoning 121001, P.R. China; 2 Liaoning Province Key Laboratory of Medical Tissue Engineering, The First Affiliated Hospital, Liaoning Medical University, Jinzhou, Liaoning 121001, P.R. China; 3 Department of Gynecology, The First Affiliated Hospital, Liaoning Medical University, Jinzhou, Liaoning 121001, P.R. China

**Keywords:** gastric stromal tumor, tissue factor pathway inhibitor-2, invasion, metastasis

## Abstract

The aim of this study was to explore the expression of tissue factor pathway inhibitor-2 (TFPI-2) in gastric stromal tissue and its clinical significance. TFPI-2 expression was detected by immunohistochemical analysis, RT-PCR and western blotting in tumor, peritumoral and gastric normal tissues from 72 patients with gastric stromal tumors. The level of TFPI-2 expression was observed to be significantly higher in gastric normal tissue than in peritumoral tissue, and was significantly higher in peritumoral tissue than in tumor tissue (P<0.01). As the NIH grade increased, the level of TFPI-2 expression decreased (P<0.01). A low expression level of TFPI-2 was closely associated with invasion and metastasis of gastric stromal tumors. In conclusion, the level of TFPI-2 expression was higher in gastric normal tissue than in gastric stromal tumors. Low expression levels of TFPI-2 may be associated with invasion and metastasis of gastric stromal tumors.

## Introduction

Gastrointestinal stromal tumors (GISTs), a type of common gastrointestinal mesenchymal tumor, account for 1–4% of gastroenteric tumors and 60% of GISTs occur in the stomach. Clinical features, pathology, immunophenotype and genetic features of GISTs have been elucidated, but the pathogenesis, biological behaviors and treatment remain unclear (1). Therefore, the control of tumor invasion and metastasis remains a major challenge in the treatment of GIST (2,3).

Tissue factor pathway inhibitor-2 (TFPI-2) expression is downregulated in a variety of tumors, and is associated with tumor invasion and metastasis (4,5). In the present study, TFPI-2 expression was detected in gastric stromal tumors using immunohistochemistry, RT-PCR and western blotting, and the correlation of TFPI-2 expression with the invasion and metastasis of gastric stromal tumors was explored. The results are likely to provide novel insight into the treatment of gastric stromal tumors.

## Materials and methods

All study methods were approved by the Ethics Committee of the First Affiliated Hospital, Liaoning Medical University (Jinzhou, China). All the subjects enrolled into the study provided written formal consent prior to participation.

### Clinical data

Between January 2006 and April 2012, 72 patients presenting with gastric stromal tumors were enrolled in this study. Of the 72 patients, 35 were men and 37 were women, with a median age of 56.4 years (range, 33–78 years). During surgery, 72 samples of tumor tissue, peritumoral tissue (within 3 cm of the tumor edge) and gastric normal tissue (3 cm from the tumor edge) were collected from the 72 patients and stored at −80°C for future use. The diagnostic criteria for gastric stromal tumor were as follows: i) Conventional morphology is in line with GIST characteristics and immunohistochemical analysis indicates positive CD117 staining; ii) conventional morphology is in line with CIST characteristics, although CD117 is negative, there is c-kit or PDGFRA gene mutation; iii) conventional morphology is in line with CIST characteristics, although CD117 is negative and there is no c-kit or PDGFRA gene mutation, other tumors including smooth muscle tumor, fibromatosis and neurogenic tumor may be excluded (6). Gastric stromal tumors in this study were evaluated according to NIH grading standards established by Fletcher *et al*(7) based on GIST biological behaviors. Tumors with a diameter of <2 cm and karyokinesis ≤5/50 high power fields (HPF) were classed as a very low-invasion risk (grade I); tumors with a diameter of ≥2,<5 cm and karyokinesis ≤5/50 HPF were classed as a low-invasion risk (grade II); tumors with a diameter of ≥5,<10 cm and karyokinesis ≤5/50 HPF or tumors with a diameter <5 cm and karyokinesis of 6–10/50 HPF were classed as a moderate-invasion risk (grade III); tumors with a diameter ≥5 cm and karyokinesis >5/50 HPF or tumors with a diameter ≥10 cm or karyokinesis >10/50 HPF were classified as a high-invasion risk (grade IV).

### Detection of TFPI-2 protein expression by immunohistochemistry

The samples were embedded in paraffin, sectioned at a thickness of 4 μm and stained with immunohistochemistry S-P kit provided by Beijing Bioss Biosynthesis Biotechnology Co., Ltd. The primary antibody of TFPI-2, provided by Beijing Biosynthesis Biotechnology Co., Ltd., was diluted to 1:400. The scoring for the immunostaining intensity of positive cells was as follows: 0, colorless; 1, light yellow; and 2, brown-yellow or brown. The scoring for the percentage of positive cells was as follows: 0, <5%; 1, 5–9%; 2, 10–19%; 3, 20–49%; and 4, ≥50%. Immunohistochemical staining index = score from the rate of positive cells × score from staining intensity of positive cells. Immunohistochemical staining index scores of ≤2 were regarded as negative (−) and scores of ≥3 as positive (+).

### RNA extraction

The tissue was made into powders and then placed in an Eppendorf tube. TRIzol fluid was added at a ratio of 1 ml (trizol fluid) : 100 mg (tissue). After homogenization, 0.2 ml of chloroform was added followed by centrifugation at 3,800 × g for 15 min. The supernatant was placed in another Eppendorf tube and then 0.5 ml of isopropanol was added. Ten minutes later, the tube underwent centrifugation at 3,800 × g for 15 min followed by removal of supernatant.

### Detection of TFPI-2 mRNA expression by RT-PCR

Total RNA was extracted from each sample and 2 μg total RNA was used in RT-PCR. The upstream and downstream primers of TFPI-2 and β-actin were synthesized by Sangon Co., Ltd (Shanghai, China). The upstream and downstream primers of TFPI-2 were 5′-TCTGCCAATGTGACTCGCTAT-3′ and 5′-TATTGTCATTCCCTCCACAGC-3′, respectively, with a synthetic product length of 88 bp. The upstream and downstream primers of β-actin were 5′-CTGGGACGACATGGAGAAAA-3′ and 5′-AAGGAAGGCTGGAAGAGTGC-3′, respectively, with a synthetic product length of 216 bp. RT-PCR conditions were as follows: reverse transcription at 50°C for 30 min, inactivation of reverse transcriptase at 94°C for 2 min, denaturing at 94°C for 30 sec, reannealing at 56°C for 30 sec and elongation at 72°C for 1 min, 30 cycles. Finally, the PCR products were subjected to 2% agarose gel electrophoresis.

### Detection of TFPI-2 protein expression by western blotting

The samples (200 mg) were placed into a homogenizer and cut into small pieces. Thirty minutes after the addition of 400 μl protein lysate, the sample was centrifuged at 3,800 × g for 5 min. The supernatant was placed in a 0.5-ml centrifuge tube and boiled for 5 min following the addition of buffer. The supernatant underwent SDS-PAGE (stacking gel with 80 V and separating gel with 150 V) and was transferred onto a 0.45 μm-PVDF membrane followed by sealing with 5% dried skimmed milk for one hour. Rabbit anti-human TFPI-2 and β-actin antibodies (Beijing Bioss Biosynthesis Biotechnology Co., Ltd.; Beijing, China) were added at 4°C overnight. After washing the membrane, horseradish peroxidase-labeled secondary antibodies (1:1,000; Beijing Bioss Biosynthesis Biotechnology Co., Ltd.) were added at room temperature for 60 min. The membrane was then washed, and coloration was performed. Coloration was performed with an enhanced chemiluminescence detection kit purchase from BestBio (Shanghai, China). The levels of protein expression were quantified through the fluorescent scanning for blotting membrane and analysis of molecular weights and optical density values of target bands with gel image processing system (Syngene, Frederick, MD, USA).

### Statistical analysis

Statistical analysis was performed with SPSS version 16.0 software (SPSS, Inc., Chicago, IL, USA). Measurement data were expressed as mean ± SD. A Student’s t-test was used in comparisons between two groups and one-factor analysis of variance was used in comparisons among multiple groups. A χ^2^ test was used in comparisons between enumeration data. P<0.05 was considered to indicate a statistically significant result.

## Results

### Immunohistochemical staining

**Figure 1:**
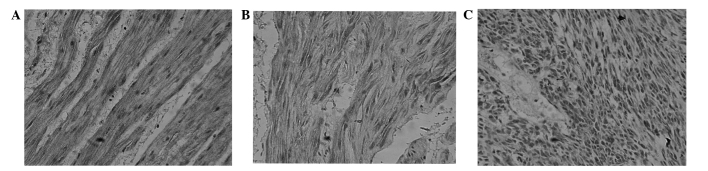
Immunohistochemical staining with SP method under Olympus optical microscope. TFPI-2 protein expression in (A) gastric normal tissue, (B) TFPI-2 peritumoral tissue and (C) tumor tissue (magnification, ×400). TFPI-2, tissue factor pathway inhibitor-2.

TFPI-2 protein was mainly located in the cytoplasm as brown or yellow stained particles. TFPI-2 protein expression was present in the tumor, peritumoral and gastric normal tissues, but TFPI-2 protein expression was stronger in gastric normal tissue than in peritumoral tissue and tumor tissue, and stronger in peritumoral tissue than in gastric normal tissue (Fig. 1). No significant difference was identified in the frequency of a negative TFPI-2 immunohistochemical staining result between male and female patients or between different age groups, but there was a statistically significant difference between the patients with and without tumor invasion or metastasis (P<0.05, Table I).

### TFPI-2 mRNA expression

**Figure 2:**
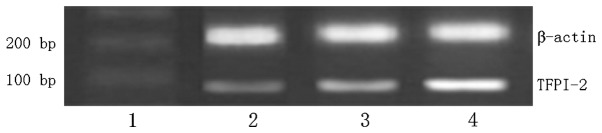
TFPI-2 mRNA expression in tumor, peritumoral and gastric normal tissues. Lane 1, marker; Lane 2, tumor tissue; Lane 3, peritumoral tissue; Lane 4, gastric normal tissue. TFPI-2, tissue factor pathway inhibitor-2.

The expression level of TFPI-2 mRNA was significantly increased in gastric normal tissue and peritumoral tissue compared with that in tumor tissue (P<0.01; Fig. 2 and Table II). As the NIH grade increased, TFPI-2 mRNA expression was downregulated (P<0.01). No statistically significant difference was identified in TFPI-2 mRNA expression between male and female patients, or between different age groups. However, there was a statistically significant difference in the level of TFPI-2 mRNA expression between the patients with and without tumor invasion or metastasis (P<0.05; Table III).

### TFPI-2 protein expression

**Figure 3:**
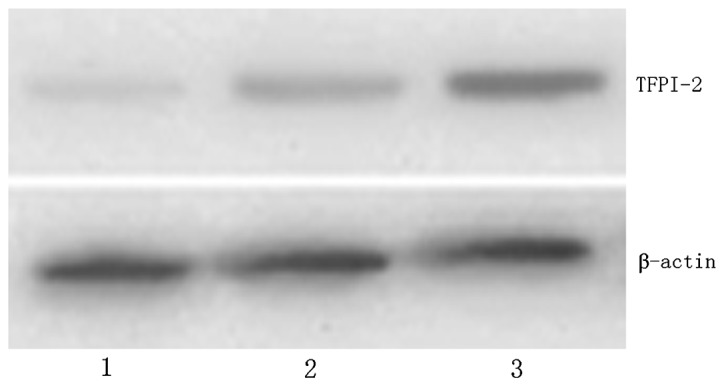
TFPI-2 protein expression in tumor, peritumoral and gastric normal tissues. Lane 1, tumor tissue; Lane 2, peritumoral tissue; Lane 3, gastric normal tissue. TFPI-2, tissue factor pathway inhibitor-2.

The expression level of TFPI-2 protein was significantly increased in gastric normal and peritumoral tissues compared with that in tumor tissue, (P<0.01; Fig. 3 and Table II). With the increase in NIH grade, TFPI-2 protein expression was downregulated (P<0.01). No statistically significant difference was identified in TFPI-2 protein expression between male and female patients, or between different age groups. However, there was a statistically significant difference in the level of TFPI-2 protein expression between the patients with and without tumor invasion or metastasis (P<0.05; Table III).

## Discussion

At present, the evaluation of the degree of malignancy in GIST is mainly based on tumor size and the number of karyokineses. In the present study, according to the GIST grading scheme established by Fletcher *et al*(7), gastric stromal tumors were divided into the categories of very low-invasion risk (grade I), low-invasion risk (grade II), moderate-invasion risk (grade III) and high-invasion risk (grade IV). The results indicated that there was a significant difference in TFPI-2 expression between tumor, peritumoral and gastric normal tissues, and between tumors of different grades. TFPI-2 expression was observed to be significantly decreased in the patients with tumor invasion or metastasis.

TFPI-2, a type of serine proteinase inhibitor, effectively inhibits the activities of numerous proteolytic enzymes, including matrix metalloproteinases, fibrinogenase, trypsin, chymotrypsin and cathepsin. TFPI-2 is able to inhibit the growth, invasion and metastasis of glioma (8), lung cancer (9), prostate cancer (10), laryngeal cancer (11) and pancreatic cancer (12).

The results of the present study indicate that TFPI-2 expression was inhibited in the gastric tumor and peritumoral tissues, suggesting that the inhibition of TFPI-2 expression may decrease the stability of the extracellular matrix. The results also indicated that as the NIH grade increased, the level of TFPI-2 expression was decreased, and a statistically significant difference was identified in TFPI-2 expression levels between tumors with and without invasion or metastasis. This suggests that low expression of TFPI-2 may promote the growth, invasion or metastasis of gastric stromal tumors with a poor prognosis. A possible mechanism of action for TFPI-2 protein, the TFPI-2 gene expression product, is inhibition of the activities of numerous proteolytic enzymes and a subsequent reduction of their damaging effects on the extracellular matrix. Proteolytic enzymes secreted by tumor cells are involved in the degradation of the extracellular matrix, which is the key step of tumor invasion or metastasis. A previous study indicated that TFPI-2 gene expression inhibits the growth of choriocarcinoma by inducing choriocarcinoma cell apoptosis (13). The mechanism by which TFPI-2 expression inhibits gastric stromal tumors requires further study.

In summary, the TFPI-2 gene may play an important role in the invasion and metastasis of gastric stromal tumors. This finding is likely to prompt novel ideas for judging the degree of malignancy of gastric stromal tumors, predicting invasion or metastasis and evaluating the prognosis of patients with gastric stromal tumors.

## Figures and Tables

**Table I:** Correlation between TFPI-2 protein expression and clinical features of gastric stromal tumor.

		TFPI-2 protein expression, n (%)			
				
Characteristics	n	Negative	Positive	χ^2^	P-value
Gender					
Male	35	23 (65.71)	12 (34.29)	0.45	0.50
Female	37	27 (72.97)	10 (27.03)		
Age (years)					
<55	27	20 (74.07)	7 (25.93)	0.44	0.51
≥55	45	30 (66.67)	15 (33.33)		
Invasion or metastasis					
Yes	20	18 (90.00)	2 (10.00)^a^	5.51	0.02
No	52	32 (61.54)	20 (38.46)		

a P<0.05, compared with the patients without tumor invasion and metastasis.

TFPI-2, tissue factor pathway inhibitor-2.

**Table II:** mRNA and protein expression of TFPI-2 in tumor, peritumoral and gastric normal tissues.

Tissue (n=72)	TFPI-2 mRNA expression (relative OD value)	F-value	P-value	TFPI-2 protein expression (relative OD value)	F-value	P-value
Tumor	0.3943±0.1046^a^,^b^	18.15	<0.01	0.2363±0.0890^a^,^b^	17.56	<0.01
Peritumoral	0.5479±0.0871^a^			0.3795±0.0494^a^		
Normal	0.6386±0.1023			0.4927±0.0606		

a P<0.01, compared with gastric normal tissue;

b P<0.01, compared with peritumoral tissue.

TFPI-2, tissue factor pathway inhibitor-2; OD, optical density.

**Table III:** Correlation of TFPI-2 expression in tumor tissue with clinicopathologic characteristics.

Characteristics	n	TFPI-2 mRNA expression (relative OD value)	t- or F-value	P-value	TFPI-2 protein expression (relative OD value)	t- or F-value	P-value
Gender							
Male	35	0.3698±0.1246	1.027	0.32	0.2189±0.0899	0.837	0.41
Female	37	0.4175±0.0753			0.2528±0.0844		
Age (years)							
<55	27	0.4038±0.1151	0.303	0.76	0.2642±0.0880	1.146	0.27
≥55	45	0.3886±0.1026			0.2196±0.0879		
NIH grade							
I	20	0.5231±0.0581	15.418	<0.01	0.3438±0.0482	14.706	<0.01
II	12	0.4429±0.0599			0.2961±0.0381		
III	9	0.3705±0.0764^a^,^b^			0.2020±0.0453^a^,^b^		
IV	31	0.2993±0.0437^a^,^c^			0.1538±0.0466^a^,^c^		
Invasion or metastasis							
Yes	20	0.3236±0.0627^d^	2.779	0.01	0.1825±0.0472^d^	2.368	0.03
No	52	0.4215±0.1012			0.2570±0.0968		

a P<0.01, compared with grade I;

b P<0.05 and

c P<0.01, compared with grade II;

d P<0.05, compared with no mucosal invasion.

TFPI-2, tissue factor pathway inhibitor-2; OD, optical density.
